# Altered ocular surface microbiota in obesity: a case-control study

**DOI:** 10.3389/fcimb.2024.1356197

**Published:** 2024-03-12

**Authors:** Chenghong Liang, Limin Wang, Xiudan Wang, Yifan Jia, Qinyuan Xie, Lingyun Zhao, Huijuan Yuan

**Affiliations:** ^1^ Department of Endocrinology, Zhengzhou University People’s Hospital, Zhengzhou, China; ^2^ Department of Endocrinology, Henan Provincial People’s Hospital, Zhengzhou, China

**Keywords:** obesity, ocular surface microbiota, cytochrome P450, oligomerization domain-like receptor, lipid metabolism

## Abstract

**Purpose:**

This study aimed to investigate the composition of ocular surface microbiota in patients with obesity.

**Methods:**

This case-control study, spanning from November 2020 to March 2021 at Henan Provincial People’s Hospital, involved 35 patients with obesity and an equivalent number of age and gender-matched healthy controls. By employing 16S rRNA sequencing, this study analyzed the differences in ocular surface microbiota between the two groups. The functional prediction analysis of the ocular surface microbiota was conducted using PICRUSt2.

**Results:**

The alpha diversity showed no notable differences in the richness or evenness of the ocular surface microbiota when comparing patients with obesity to healthy controls (Shannon index, *P*=0.1003). However, beta diversity highlighted significant variances in the microbiota composition of these two groups (ANOSIM, *P*=0.005). LEfSe analysis revealed that the relative abundances of *Delftia*, *Cutibacterium*, *Aquabacterium*, *Acidovorax*, *Caulobacteraceae unclassified*, *Comamonas* and *Porphyromonas* in patients with obesity were significantly increased (*P*<0.05). Predictive analysis using PICRUSt2 highlighted a significant enhancement in certain metabolic pathways in patients with obesity, notably xenobiotics metabolism via cytochrome P450 (CYP450), lipid metabolism, and the oligomerization domain (NOD)-like receptor signaling pathway (*P*<0.05).

**Conclusions:**

Patients with obesity exhibit a distinct ocular surface core microbiome. The observed variations in this microbiome may correlate with increased activity in CYP450, changes in lipid metabolism, and alterations in NOD-like receptor signaling pathways.

## 1 Introduction

Obesity, characterized as a chronic, multifactorial disease, has escalated into a worldwide health crisis, impacting around 641 million individuals globally (NCD-RisC, 2016). It is widely recognized for increasing the risk of numerous health issues including metabolic syndrome, cardiovascular diseases, type 2 diabetes, and hypertension, posing a significant public health challenge (De Andrade Mesquita et al., 2023). Despite the scarcity of research on obesity’s potential effects on eye diseases, it’s crucial to acknowledge its potential impact in this area as well.

Severe obesity may induce irreversible blindness by promoting the occurrence and progression of diseases such as age-related macular degeneration (AMD) and glaucoma. Obesity, particularly when induced early in life, can lead to long-term pro-inflammatory changes in monocytes (Hata et al., 2023). This inflammation exacerbates pathological conditions such as retinal neovascularization and neuron degeneration, closely associated with vision loss and AMD-related blindness (Hata et al., 2023). Furthermore, obesity-related increases in orbital fat, along with decreased choroidal perfusion and ocular blood flow, might lead to higher intraocular pressure, fostering the development and progression of glaucoma (Pezzino et al., 2023). In addition to these factors, blood lipid abnormalities may cause dysfunction of the meibomian glands by disrupting the rheological properties of the eyelid fat or blocking the meibomian gland (Osae et al., 2019). The systemic chronic inflammation, elevated leptin levels, and oxidative stress associated with obesity are also potential contributors to various eye diseases (Cheung and Wong, 2007; Hata et al., 2023). Moreover, Hata et al. discovered that obesity’s effects on the eyes might be lasting, irreparable even with subsequent weight loss (Hata et al., 2023).

The relationship between the gut microbiota and obesity is unmistakable. Regarding obesity-related eye diseases, studies have discovered that the gut microbiota may contribute by inducing increased intestinal permeability and lipopolysaccharide translocation in HFD-fed mice, leading to laser-induced choroidal inflammation and neovascularization (Andriessen et al., 2016). Interestingly, treatment with the lipid-lowering drug fenofibrate in HFD-fed mice has been observed to reduce retinal inflammation and increase the abundance of gut microbiota that produce short-chain fatty acids (SCFAs), offering potential therapeutic insights (Wang et al., 2022). However, with the Human Microbiome Project dramatically broadening the scope of microbiome research to include various ecological niches beyond traditional domains like the gut and oral cavity, the pivotal roles of localized microbiomes, including those on the ocular surface, within their specific environments are garnering more focus (iHMP Research Network Consortium, 2019).

Current research underscores the essential role of the ocular surface microbiome in maintaining ocular homeostasis (Gomes JÁ et al., 2020; Deng et al., 2021; Herzog et al., 2023; Zhao et al., 2023). It achieves this through key mechanisms such as selective immune tolerance (Ueta and Kinoshita, 2010) and the resistance to pathogenic colonization (St Leger et al., 2017), all while being modulated by both local ocular conditions and systemic health factors (Gomes JÁ et al., 2020). Multiple studies have shown that epithelial cells of the cornea and conjunctiva can selectively produce pro-inflammatory factors to respond to specific pathogenic bacteria on the ocular surface without causing inflammation to the commensal bacteria (Cavuoto et al., 2019). Changes in the ocular surface microbiota are closely related to AMD, glaucoma, dry eye syndrome, meibomian gland dysfunction, conjunctivitis, keratitis, and diabetes (Li et al., 2019; Gomes JÁ et al., 2020; Deng et al., 2021).

Similar to the alterations observed in the gut microbiota, obesity-associated chronic low-grade inflammation may also influence the ocular surface microbiota. This can potentially compromise the eye’s protective barrier, thus affecting ocular health through mechanisms that extend beyond well-known systemic pathways. Despite the recognized importance of the ocular surface microbiota in maintaining eye health, the specific changes it undergoes in patients with obesity have yet to be fully investigated. Considering the ocular surface’s accessibility and the pivotal role its microbiome plays in preserving ocular equilibrium, our study seeks to delve into these alterations. We aim to provide new perspectives in ocular surface microbiota research, contributing to the development of strategies for managing and preventing obesity-related eye diseases.

## 2 Materials and methods

### 2.1 Clinical study design

This case-control study approved by the Ethics Committee of Henan Provincial People’s Hospital (Eth.2020113). Written informed consents were obtained from all subjects for this study.

From November 2020 to March 2021, 35 patients diagnosed with obesity were recruited at the Endocrinology Obesity Clinic of Henan Provincial People’s Hospital. The control group consisted of 35 healthy volunteers who underwent annual physical examinations at the same hospital, matched with the patients with obesity in terms of age and gender.

The inclusion criteria for patients with obesity were as follows: (1) BMI ≥ 28 kg/m^2^; (2) absence of ocular conditions such as AMD, dry eye, meibomian gland dysfunction, trachoma, conjunctivitis, keratitis, glaucoma, cataracts, and significant myopia; (3) freedom from serious systemic illnesses including heart failure, coronary artery disease, stroke, renal insufficiency (acute or chronic), liver cirrhosis, and malignancies; (4) an age range of 18 to 59 years.

The inclusion criteria for healthy controls included: (1) 18.5 kg/m^2^ < BMI < 23.9 kg/m^2^; (2) adherence to criteria numbers 2 through 4 specified for patients with obesity.

For both patients with obesity and healthy controls, the exclusion criteria were: (1) a history of eye surgery; (2) administration of antibiotics or corticosteroids within the previous 6 months; (3) usage of eye drops in the past 6 months; (4) current contact lens wearers; (5) current pregnancy or lactation; (6) presence of diabetes or immune system disorders.

### 2.2 Clinical data collection

We collected basic information from the subjects through a questionnaire, including gender, age, medication history in the past six months, history of systemic or ocular diseases, history of eye surgery, and contact lens use. The measurement of the subjects’ height, weight, waist and hip circumference, and blood pressure was conducted in the morning while the subjects were fasting. The clinical parameters of subjects, including triglycerides (TG), total cholesterol (TC), alanine transaminase (ALT), aspartate transaminase (AST), creatinine (Cr), uric acid (UA), and fasting plasma glucose (FPG), were retrieved from the Henan Provincial People’s Hospital’s electronic medical record system.

### 2.3 Sample collection

The collection of ocular surface microbiome samples was conducted prior to the physical examination, exclusively by the same experimenter who had received professional training in aseptic techniques, to maximally avoid potential errors and contamination during the sampling process. For sampling, 0.4% oxybuprocaine hydrochloride eye drops were first used for local anesthesia of the ocular surface. Then, using a disposable sterile cotton swab, samples of the ocular surface microbiome were collected from the subconjunctival space. After collection, the swabs were placed individually in 1.5 mL Eppendorf sterilized tubes and stored in a -80°C freezer within 10 minutes until analysis. Additionally, during the sample collection process, we utilized sterile swabs containing the topical anesthetic as an environmental blank control to further reduce the risk of sample contamination.

### 2.4 DNA extraction, PCR amplification, and sequencing

All eye surface samples underwent identical DNA extraction and PCR amplification processes by the same laboratory team. The samples were mixed with a lysis buffer and glass beads (BioSpec Products, Inc., USA), vortexed, and heated at 70°C for an hour. Bead beating followed for DNA extraction using the E.Z.N.A.^®^ Stool DNA Kit (Omega Bio-tek, Inc., GA).

For amplifying the V3-V4 region of 16S rRNA genes, primers F1 (5′-CCTACGGGNGGCWGCAG-3′) and R2 (5′-GACTACHVGGGTATCTAATCC-3′), corresponding to positions 341 to 805 in the *Escherichia coli* 16S rRNA gene, were used. PCR was performed in an EasyCycler 96 PCR system (Analytik Jena Corp., AG) with a specific program. The PCR products from various samples were indexed, mixed equally, and sequenced using the Miseq platform (Illumina Inc., USA).

In the experimental phase, stringent measures were taken to minimize potential contamination. The sterilization of the environment with ultraviolet light, coupled with the use of sterile consumables and the conduct of experiments such as PCR on a super-clean workbench, further reinforced our control measures. Moreover, we established negative controls during the critical stages of DNA extraction and PCR amplification. These controls were not only set up but also sequenced alongside the samples, providing a direct comparison to identify and quantify any incidental environmental bacterial sequences.

Crucially, in the event that environmental bacterial sequences were detected in the negative controls, these sequences were systematically removed from the sample sequencing data. This step was based on the sequencing results of the negative controls and was executed to eliminate the influence of environmental bacteria on our analyses. This procedure ensured that our data analysis and subsequent interpretations were not compromised by external contamination.

### 2.5 Sequencing data analysis

The sequencing and data processing involved extracting clean data from raw data using USEARCH (version 11.0.667). Data purification was meticulously conducted from the raw dataset employing USEARCH (version 11.0.667), adhering to a stringent set of criteria to ensure integrity and quality: (1) Sequences from each sample were meticulously extracted utilizing their respective indexes, allowing no discrepancies. (2) Sequences exhibiting overlaps shorter than 16 base pairs were systematically excluded. (3) Overlaps with an error rate exceeding 0.1 were rigorously discarded to maintain data accuracy. (4) Post-merge, sequences with a length below 400 base pairs were eliminated, ensuring only data of substantial genetic information were retained. These sequences were then clustered and sorted for abundance to identify representative sequences using the UPARSE OTU analysis pipeline, excluding singletons. Operational taxonomic units (OTUs) were classified using UPARSE (version 7.1), based on 97% similarity and after removing chimeric sequences. Each OTU considered a microbial species. Annotations were made with the SILVA reference database (SSU138) (Quast et al., 2013).

Alpha diversity serves as a key indicator of the microbial communities’ richness and diversity. We employed Mothur (version 1.42.1) to measure these attributes, utilizing the ACE index to gauge richness, and the Shannon-Wiener and Simpson indices to appraise diversity. Beta diversity serves as a critical measure for comparing microbial community compositions among various groups, highlighting the differences in species distribution. We leveraged Bray-Curtis-based Principal Coordinates Analysis (PCoA) to quantify these variations through statistical distances, thus facilitating a detailed comparative analysis of the microbial community compositions within our samples. This analytical procedure was executed utilizing R version 3.6.0 (http://www.R-project.org/) and the statistical significance of the divisions was analyzed using ANOSIM test. The linear discriminant analysis (LDA) effect size (LEfSe) method (version 1.1, https://github.com/SegataLab/lefse) identified differentially abundant taxa among groups (Chen et al., 2015), and PICRUSt2 (version 2.4.1, https://github.com/picrust/picrust2) predicted functional abundances from 16S rRNA gene sequences (Douglas et al., 2020).

The Sequence Read Archive database houses the raw sequencing data and related information for the 16S rRNA gene V3-V4 regions, accessible under accession number PRJNA1044261.

### 2.6 Statistical analysis

Statistical analysis was performed using SPSS (version 26.0, Inc, Chicago, IL). Data normality was assessed with the *Shapiro-Wilk* test. Normally distributed continuous data were presented as mean ± standard deviation (SD) and analyzed using independent sample *t*-test, whereas non-normally distributed continuous data were expressed as median (interquartile range) and analyzed using the Mann-Whitney *U* test. Frequency (%) was used to present count data, and the *Fisher’s* exact test was applied for analysis. A *P*-value < 0.05 was considered statistically significant.

## 3 Results

### 3.1 Clinical parameters

This study involved 35 patients with obesity (obese group) and 35 healthy controls (healthy group). In obese group, the male-to-female ratio was 17:18, with a mean age of (36.49 ± 9.16) years and a median BMI of [32.25 (29.78, 35.67)] kg/m^2^. The male-to-female ratio in the healthy group was 19:16, with a mean age of (39.91 ± 9.25) years and a median BMI of [20.60 (19.70, 22.10)] kg/m^2^. Significant differences existed between the two groups in BMI, waist-to-hip ratio (WHR), TC, TG, and UA (*P*<0.05), but not in gender, age, ALT, AST, Cr, and FPG (*P*>0.05) (**Table 1**). The clinical parameter data indicated that both the obese and healthy groups in this study were well-represented.

**Table 1:** Characteristics of obese group and healthy group.

Characteristic	Obese group (*n*=35)	Healthy group (*n*=35)	*P value*
Age (years)	36.49 ± 9.16	39.91 ± 9.25	0.124
Gender [n (%)]			0.811
Male	17 (48.57)	19 (54.29)	
Female	18 (51.43)	16 (45.71)	
BMI (kg/m^2^)	32.25 (29.78, 35.67)	20.60 (19.70, 22.10)	<0.001
WHR	1.03 (1.01, 1.05)	0.82 (0.79, 0.84)	<0.001
TC (mmol/L)	5.66 (5.36, 6.09)	4.17 (3.50, 4.35)	<0.001
TG (mmol/L)	2.16 ± 0.97	1.20 ± 0.52	<0.001
ALT (U/L)	21.10 (14.40, 25.70)	16.40 (14.50, 22.70)	0.238
AST (U/L)	18.60 (15.60, 24.40)	21.20 (18.20, 25.20)	0.053
Urea (mmol/L)	4.53 ± 0.74	4.25 ± 0.90	0.168
Cr (μmol/L)	55.89 ± 12.74	57.19 ± 15.81	0.705
UA (μmol/L)	363.17 ± 88.02	310.91 ± 91.64	0.018
FPG (mmol/L)	5.00 (4.46, 5.30)	5.10 (4.80, 5.40)	0.317

Continuous data were presented and analyzed accordingly: normally distributed data as mean ± SD using the independent sample t-test, and non-normally distributed data as median (interquartile range) using the Mann-Whitney U test. Count data were expressed as frequency (%) and analyzed with the Fisher’s exact test. BMI, body mass index; WHR, waist-hip ratio; TC, total cholesterol; TG, triglyceride; ALT, alanine transaminase, AST, aspartate transaminase; Cr, creatinine; UA, uric acid; FPG, fasting plasma glucose.

### 3.2 Obese group had a different ocular surface microbiota

#### 3.2.1 Analysis of alpha and beta diversity of ocular surface microbiota

**Figure 1:**
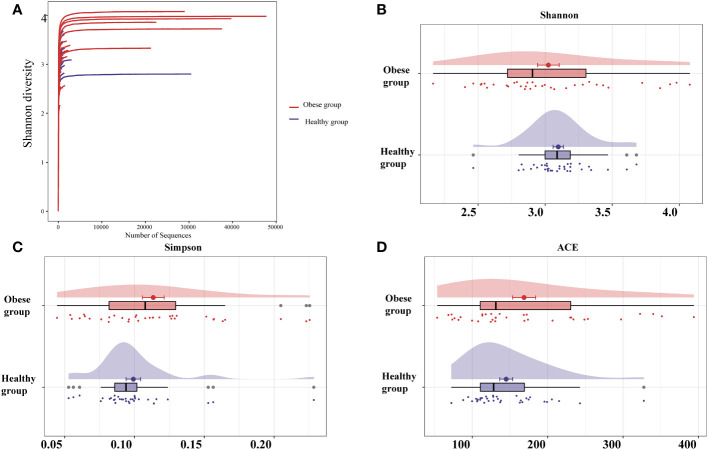
Analysis of sequencing depth and alpha diversity comparison of ocular surface microbiota between obese and healthy groups. **(A)** As the sequencing depth increases, the dilution curve progressively levels off, suggesting that the volume of sequencing data is appropriate. Indices including Shannon **(B)**, Simpson **(C)**, and ACE **(D)** revealed no significant alpha diversity differences between groups. The ACE index measures richness, Shannon and Simpson indices measure diversity.

The sequencing depth for the ocular samples was deemed adequate, as evidenced by the leveling off of the dilution curve with an increase in sequencing data (**Figure 1A**). This study analyzed the ocular surface microbiota’s richness and uniformity using the Shannon, Simpson, and ACE indices to represent alpha diversity. Notably, a trend towards higher alpha diversity was observed in the obese group, yet the differences in Shannon, Simpson, and ACE indices between the obese and healthy groups were not statistically significant (*P*>0.05) (**Figures 1B–D**; **Supplementary Table 1**).

**Figure 2:**
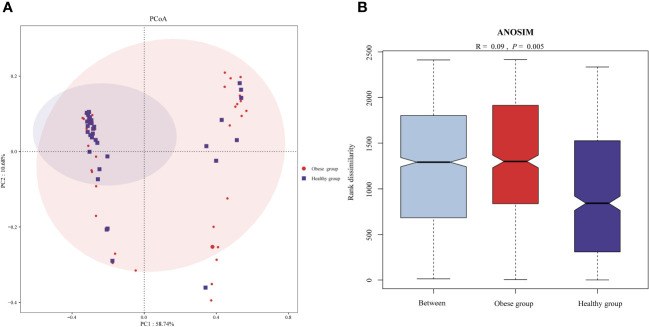
Comparison of ocular surface microbiota beta diversity between obese and healthy groups. **(A)** Principal coordinates analysis plot (based on Bray-Curtis) of the ocular surface microbiota. The separation between the ocular surface microbiota communities of the two groups was significant, suggesting distinct microbial compositions within each group. **(B)** ANOSIM analysis confirmed the difference of beta diversity.

To assess the beta diversity of the ocular surface microbiota, PCoA based on the Bray-Curtis distance was utilized, aiming to highlight variations in species abundance distribution between the obese and healthy groups. This analysis revealed a distinct separation trend in the microbiota composition of the two groups (**Figure 2A**). Further, statistical evaluation via ANOSIM confirmed the significance of this difference (*P*=0.005) (**Figure 2B**).

#### 3.2.2 Analysis of species composition of ocular surface microbiota

**Figure 3:**
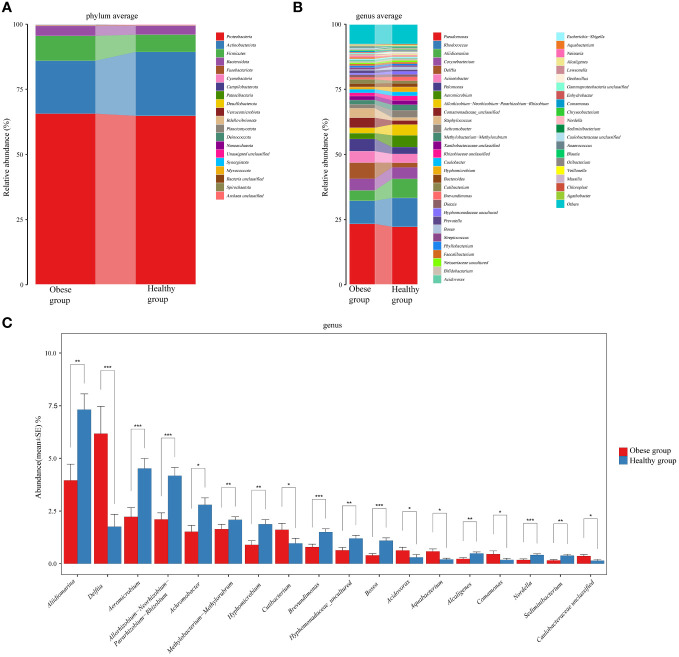
Comparative analysis of the species composition of ocular surface microbiota between obese and healthy groups. **(A)** Histograms showed the average relative abundance at the phylum level. **(B)** Histograms for the average relative abundance at the genus level. **(C)** Histograms highlighted genera with significant differences between the two groups. Different colors represented different ocular surface microbiomes. Error bars represented the standard error for each group. Mann-Whitney *U* test was applied for comparisons. ^*^ *P*<0.05, ^**^ *P*<0.01, ^***^ *P*<0.001.

Each OTU is regarded as a unique microbial species. In ocular surface samples from subjects, 663 OTUs were identified. Among these, 432 OTUs were common to both groups. The obese group had 205 unique OTUs, while the healthy group had 26. At the phylum level, we consider microorganisms with a relative abundance level greater than 1% to be dominant phyla. *Proteobacteria* (65.67% vs 64.82%), *Actinobacteria* (20.31% vs 24.54%), *Firmicutes* (9.55% vs 6.63%), and *Bacteroidetes* (3.80% vs 3.41%) were dominant in both groups (The left number in parentheses indicates the obese group, the right the healthy group). There were no significant differences in these dominant phyla between the groups (*P*>0.05) (**Figure 3A**). However, within the less prevalent phyla (relative abundance level < 0.01%), levels of *Bdellovibrionota* (*P*=0.022) and *Nanoarchaeota* (*P*=0.022) were notably higher in the obese group compared to the healthy group.

At the genus level, top six common genera in the ocular surface microbiota of both groups included *Pseudomonas* (23.36% vs 22.24%), *Rhodococcus* (8.86% vs 11.06%), *Aliidiomarina* (3.95% vs 7.31%), *Corynebacterium* (4.51% vs 4.44%), *Delftia* (6.17% vs 1.76%), and *Acinetobacter* (4.40% vs 3.40%) (**Figure 3B**). Notably, the obese group showed a significant increase in *Delftia* (*P*<0.001) and a decrease in *Aliidiomarina* (*P*=0.003) compared to the healthy group (**Figure 3C**). Additionally, *Staphylococcus* (3.65% vs 1.22%), *Streptococcus* (0.74% vs 0.57%), and *Cutibacterium* (1.6% vs 0.97%), which are part of the core microbiota genera identified in healthy individuals (Delbeke et al., 2021), were ranked 11th, 25th, and 19th, respectively.

**Figure 4:**
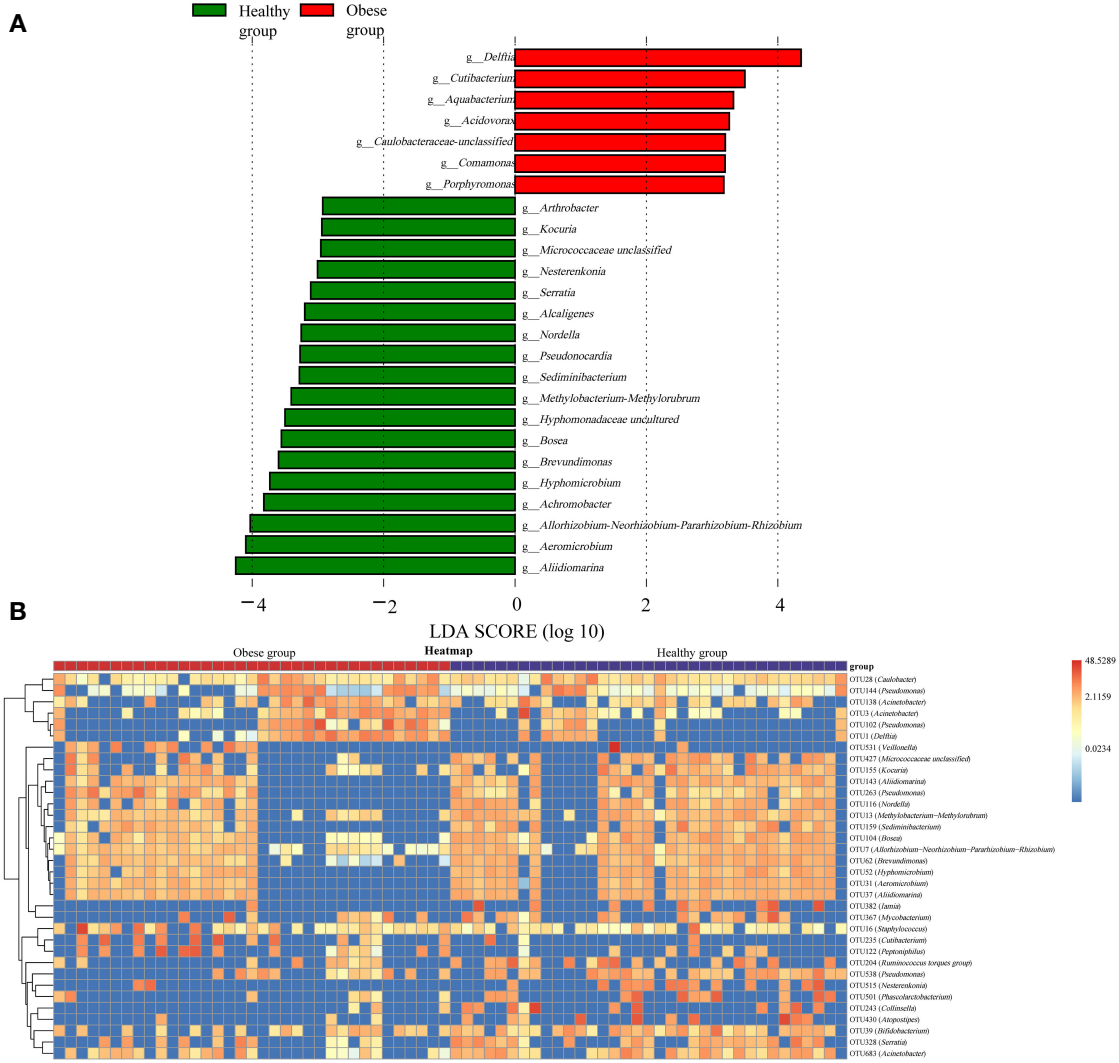
Differences in ocular surface microbiota between obese and healthy groups. **(A)** Linear discriminant analysis column diagram. The red bar graph illustrated the enhancement of ocular surface microbiota in the obese group, whereas the green bar graph highlighted the augmentation in the healthy group, with both delineations occurring at the genus level. **(B)** The random forest heatmap showed the relative abundance of the 34 OTUs significantly different in two groups. The red color indicated an increase in microbial abundance, while blue signified a decrease. The deeper the color, the greater the change in abundance.

LEfSe analysis was conducted to further explore differences in the ocular surface microbiota between obese and healthy groups. It revealed that seven genera, including *Delftia*, *Cutibacterium*, *Aquabacterium*, *Acidovorax*, *Caulobacteraceae unclassified*, *Comamonas*, and *Porphyromonas*, had a significantly higher relative abundance in the obese group (*P*<0.05). In contrast, 18 genera including *Aliidiomarina*, *Aeromicrobium*, *Allorhizobium-Neorhizobium-Pararhizobium-Rhizobium*, *Achromobacter*, *Hyphomicrobium*, *Brevundimonas*, *Bosea*, *Hyphomonadaceae uncultured*, *Methylobacterium-Methylorubrum*, *Sediminibacterium*, *Pseudonocardia*, *Nordella*, *Alcaligenes*, *Serratia*, *Nesterenkonia*, *Micrococcaceae unclassified*, *Kocuria*, and *Arthrobacter*, were significantly less abundant in the obese group (*P*<0.05) (**Figure 4A**). Additionally, a random forest analysis identified 34 OTUs with significant differences between the groups (**Figure 4B**).

#### 3.2.3 Effect of gender on ocular surface microbiota

Existing research has found that gender affects the ocular surface microbiota (Wen et al., 2017), so we also analyzed the impact of gender on the ocular microbiomes of two groups. Interestingly, we observed no significant differences in the ocular surface microbiome—neither in alpha (**Supplementary Figures 1A–C**) nor beta diversity (**Supplementary Figures 1D, E**)—between males and females among patients with obesity. The sole exception was an increased presence of the *Eubacterium coprostanoligenes group* in male patients with obesity (**Supplementary Figure 1F**). However, in healthy group, while no differences in alpha diversity were observed (**Supplementary Figures 2A-C**), significant gender-based differences in beta diversity were noted (**Supplementary Figures 2D, E**), with subsequent LefSe analysis (**Supplementary Figure 2F**) highlighting distinct variations in the ocular surface microbiota between healthy males and females.

### 3.3 Analysis of PICRUSt2 functional prediction

**Figure 5:**
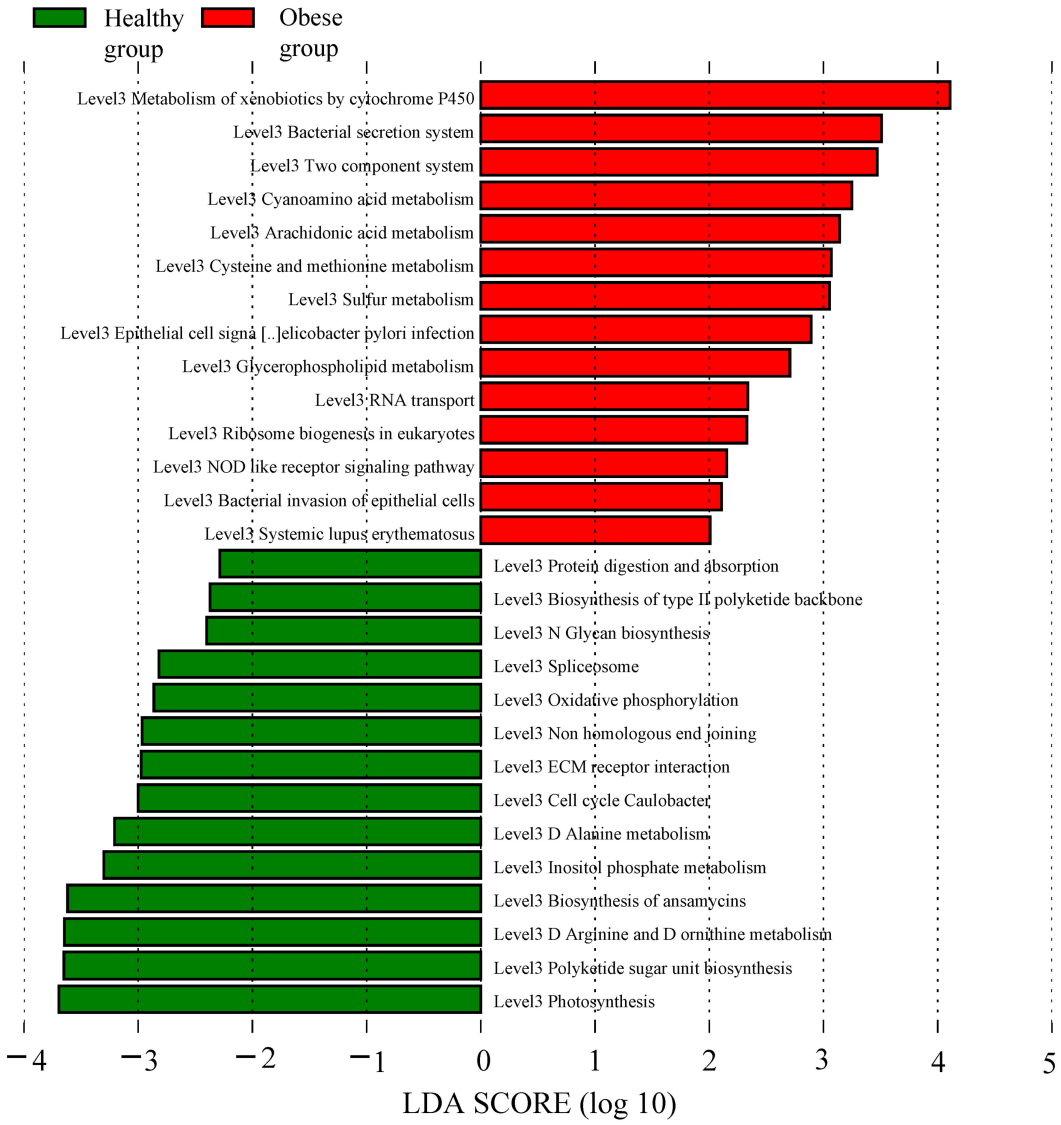
Function prediction analysis of obese and healthy groups. The red bar graph represented the increased functional metabolic pathways in the obese group, whereas the green bar graph denoted those of the healthy group.

Utilizing the KEGG pathway database, functional prediction analysis was conducted to identify significant differences in metabolic pathways (at the L3 level) between obese and healthy groups, using LEfSe analysis with stringent criteria (significant *P* values and LDA scores ≥ 2.5). The analysis revealed that several pathways were markedly enriched in obese group, including cytochrome P450 (CYP450) metabolism of xenobiotics, bacterial secretion system, cyanogenic amino acid metabolism, arachidonic acid metabolism, cysteine and methionine metabolism, sulfur metabolism, glycerophospholipid metabolism, nucleotide-binding and oligomerization domain (NOD)-like receptor signaling pathway, and bacterial invasion of epithelial cells. Conversely, pathways such as the biosynthesis of L-rhamnose-containing unit, arginine and proline metabolism, ansamycin biosynthesis, inositol phosphate metabolism, alanine metabolism, extracellular matrix (ECM)-receptor interaction, non-homologous end joining, oxidative phosphorylation, N-glycan biosynthesis, biosynthesis of type II polyketide backbone, and protein digestion and absorption showed a significant decrease in obese group (**Figure 5**).

## 4 Discussion

Previous studies employing both traditional methods and 16S rRNA sequencing, have highlighted the presence of a diverse and abundant microbial community on the human ocular surface (Dong et al., 2011; Zegans and Van Gelder, 2014; Zhou et al., 2014; Doan et al., 2016; Shin et al., 2016; Ozkan et al., 2017). These advancements have facilitated research into the ocular surface microbiota in diseases such as dry eye, meibomian gland dysfunction, keratitis, and diabetes, utilizing 16S rRNA technology (Delbeke et al., 2021). However, the composition of the ocular surface microbiota in the context of obesity remains an uncharted area. Additionally, recent studies have raised the possibility that the gut microbiota might contribute to obesity-induced choroidal pathological angiogenesis via the gut-eye axis (Andriessen et al., 2016). The role of the ocular surface microbiota in the onset and progression of obesity-related eye diseases, therefore, warrants further investigation.

In this study, we utilized 16S rRNA sequencing technology to explore the composition of the ocular surface microbiota in patients with obesity. Our analysis revealed a trend towards increased alpha diversity in the obese group compared to healthy group. Furthermore, we noted significant differences in beta diversity, particularly in the distribution of species abundance. The ocular surface microbiota predominantly consisted of the phyla *Proteobacteria*, *Actinobacteria*, *Firmicutes*, and *Bacteroidetes*. *Pseudomonas, Rhodococcus, Aliidiomarina, Corynebacterium, Delftia, and Acinetobacter* identified as core genera. Additionally, we found that *Delftia* and *Cutibacterium* were more prevalent in the microbiota of patients with obesity. Furthermore, our functional prediction analysis indicated an enrichment in pathways related to CYP450 metabolism, lipid metabolism, and NOD-like receptor signaling in the patients with obesity, alongside a decrease in the ECM receptor pathway.

Delbeke et al. reviewed 76 studies, revealing that the phylum-level ocular microbiome in healthy individuals predominantly comprises *Proteobacteria*, *Actinobacteria*, *Firmicutes*, and *Bacteroidetes*, with *Proteobacteria* and *Actinobacteria* being the most prevalent (Delbeke et al., 2021). At the genus level, the core microbiome typically includes *Staphylococcus*, *Corynebacterium*, *Propionibacterium (Cutibacterium)*, *Pseudomonas*, *Acinetobacter*, and *Streptococcus*. Our research found a similar phylum-level composition in both obese and healthy groups, suggesting a degree of stability at this taxonomic level. At the genus level, we also detected the presence of the previously mentioned microbial communities, with *Corynebacterium*, *Pseudomonas*, and *Acinetobacter* remaining as the core microbial genera in both of our study groups. Previous research has documented the presence of *Corynebacterium*, *Pseudomonas*, and *Acinetobacter* across various global populations, albeit with varying abundances (Ozkan and Willcox, 2019; Delbeke et al., 2021). *Corynebacterium mastitidis*, within the genus *Corynebacterium*, has been closely linked to the maintenance of ocular immune homeostasis (St Leger et al., 2017). Moreover, *Pseudomonas* and *Acinetobacter* have been associated with the development of conditions such as Stevens-Johnson syndrome, type 2 diabetes, and trachoma (Gomes JÁ et al., 2020). Although our study did not reveal significant differences in these three microbial genera between the groups, our findings lend additional support to their potential pivotal role in sustaining ocular homeostasis.

The ocular surface microbiota in healthy individuals is typically stable and exhibits low diversity (Ozkan and Willcox, 2019). However, in metabolic diseases such as diabetes, there is a significant increase in the alpha diversity of ocular surface microbiota (Li et al., 2019). Our study observed a trend towards increased alpha diversity and a substantial rise in OTUs among patients with obesity. Furthermore, the pronounced differences in beta diversity between patients with obesity and healthy individuals indicate a distinct ocular surface microbiota composition in those with obesity. Noteworthy fluctuations at the genus and OTUs levels, marked by an increase in *Cutibacterium* and *Delftia*, as well as the discovery of 205 unique OTUs in the obese group, further affirm the presence of a specialized core microbiome in those affected by obesity. These insights reveal that the standard makeup of the ocular surface microbiota is subject to significant modifications in light of metabolic shifts, which may jeopardize the stability of the ocular surface’s ecological balance.

Notably, the abundance of *Cutibacterium (Propionibacterium)*, previously associated with severe meibomian gland dysfunction (Watters et al., 2017) and identified as a core microbiota component in dry eye syndrome (Andersson et al., 2021), was markedly higher in patients with obesity. Considering the known link between obesity and a heightened risk of ocular conditions like meibomian gland dysfunction and dry eye syndrome (Cheung and Wong, 2007; Osae et al., 2019), our findings raise the possibility that ocular surface microbiota could play a role in the onset and progression of obesity-related ocular diseases, though further studies are necessary to establish this connection.

In their research on chronic cicatrizing conjunctivitis (CCC), Di Zazzo et al. discovered that an overactive CYP450 system might trigger inflammation in CCC by signaling lipid mediators, including arachidonic acid metabolites and lysolecithin (Di Zazzo et al., 2020). Our study’s functional predictions indicate significant enrichment of CYP450 and lipid metabolism pathways, particularly those involving arachidonic acid and glycerophospholipids, in patients with obesity. Arachidonic acid, via CYP450 metabolic pathways, can produce various signaling lipids, while glycerophospholipids can generate lysolecithin through phospholipase catalysis (Di Zazzo et al., 2020). These results imply that abnormal lipid metabolism in patients with obesity could contribute to chronic ocular inflammation mediated by CYP450. However, due to the absence of targeted metabolomics in ocular tissues and the complexity of CYP450 metabolic mechanisms, additional research is required to verify these findings.

Chronic inflammation in the eye may disrupt the ocular barrier, contributing to T cell infiltration, the loss of conjunctival goblet cells and epithelium, apoptosis, and the secretion of inflammatory cytokines. This process potentially increases vascular permeability and impairs the normal function of the ocular barrier, possibly altering the ocular surface microbiota (Mantelli and Argüeso, 2008). Our study revealed significant reductions in ECM-related pathways in patients with obesity, while pathways associated with the NOD-like receptor signaling and bacterial infections were notably enriched. The ECM, comprising the basement membrane and interstitial matrix, is vital for the adhesion, differentiation, and growth of epithelial and goblet cells, and it maintains the mechanical and structural integrity of the eye (Makuloluwa et al., 2023). Its diminished pathways indicate a compromised ocular barrier in obesity. NOD-like receptors, known for their critical role in ocular inflammation as pathogen-associated molecular pattern receptors, were also a focus of our study. An mRNA analysis of 110 patients with corneal ulcers showed heightened expression of NOD-like receptor protein 3 (NLRP3) and increased levels of inflammatory cytokines such as interleukin-1β (IL-1β), IL-8, and IL-17 (Karthikeyan et al., 2011). Similar trends in *NLRP3* gene expression and IL-1β levels were observed in patients with dry eye syndrome (Zheng et al., 2015). In addition, our study also found that the abundance of the genus *Delftia*, which is closely related to corneal infection and postoperative endophthalmitis, was relatively higher in patients with obesity (Dantam et al., 2020; Deb et al., 2020). We hypothesize that the ocular surface microbiota in obesity may exacerbate ocular inflammation through the NOD-like receptor signaling pathway, leading to a compromised ocular barrier and a heightened risk of ocular diseases. Future research should focus on enhancing the detection of ocular inflammatory cytokines and conducting ocular biopsies and animal experiments to substantiate these hypotheses.

Additionally, previous studies have found that gender also affects the ocular surface microbiome (Wen et al., 2017). Interestingly, we did not observe gender-induced microbial differences in the obese group, whereas clear differences were evident in the healthy group. These findings lead us to hypothesize that the influence of obesity on the ocular surface microbiota may outweigh gender differences. In future studies focusing on the ocular surface microbiota, it may be necessary to regard obesity as a significant factor and incorporate it into the analysis.

While this study’s significant findings provide valuable insights, it’s worth noting the limitations to better appreciate the context and potential of the research. The study’s relatively small sample size and regional focus, along with the younger age of the participants, may limit broader applicability. Additionally, the functional forecasts provided by PICRUSt2 tend to focus on established functionalities and fail to capture the full complexity of microbial communities at the strain level. However, it sets a strong foundation for future research, suggesting the benefit of larger-scale, multi-center studies that encompass diverse age groups and ethnicities to further validate and expand upon these findings. Such studies should also incorporate metagenomics and metabolomics to further validate and extend these findings. Although the study does not establish a direct causal link between ocular surface microbiota and obesity or related eye diseases, it opens new avenues for research. Future investigations, including animal experiments and mechanistic studies, are poised to build upon this groundbreaking work, delving deeper into the mechanisms at play. In essence, this study serves as a catalyst for more comprehensive research.

## Data availability statement

The datasets presented in this study can be found in online repositories. The names of the repository/repositories and accession number(s) can be found below: https://www.ncbi.nlm.nih.gov/, PRJNA1044261.

## Ethics statement

The studies involving humans were approved by Ethics Committee of Henan Provincial People’s Hospital. The studies were conducted in accordance with the local legislation and institutional requirements. The participants provided their written informed consent to participate in this study.

## Author contributions

CL: Writing – original draft, Writing – review & editing. LW: Writing – original draft, Writing – review & editing. XW: Writing – original draft, Writing – review & editing. YJ: Data curation, Writing – review & editing. QX: Writing – review & editing. LZ: Writing – review & editing. HY: Writing – original draft, Writing – review & editing.
