# Supplementary figures and images for: Altered ocular surface microbiota in obesity: a case-control study
Source: Front Cell Infect Microbiol. 2024 Mar 12;14:1356197. doi: 10.3389/fcimb.2024.1356197 (PMC10963539; doi:10.3389/fcimb.2024.1356197)

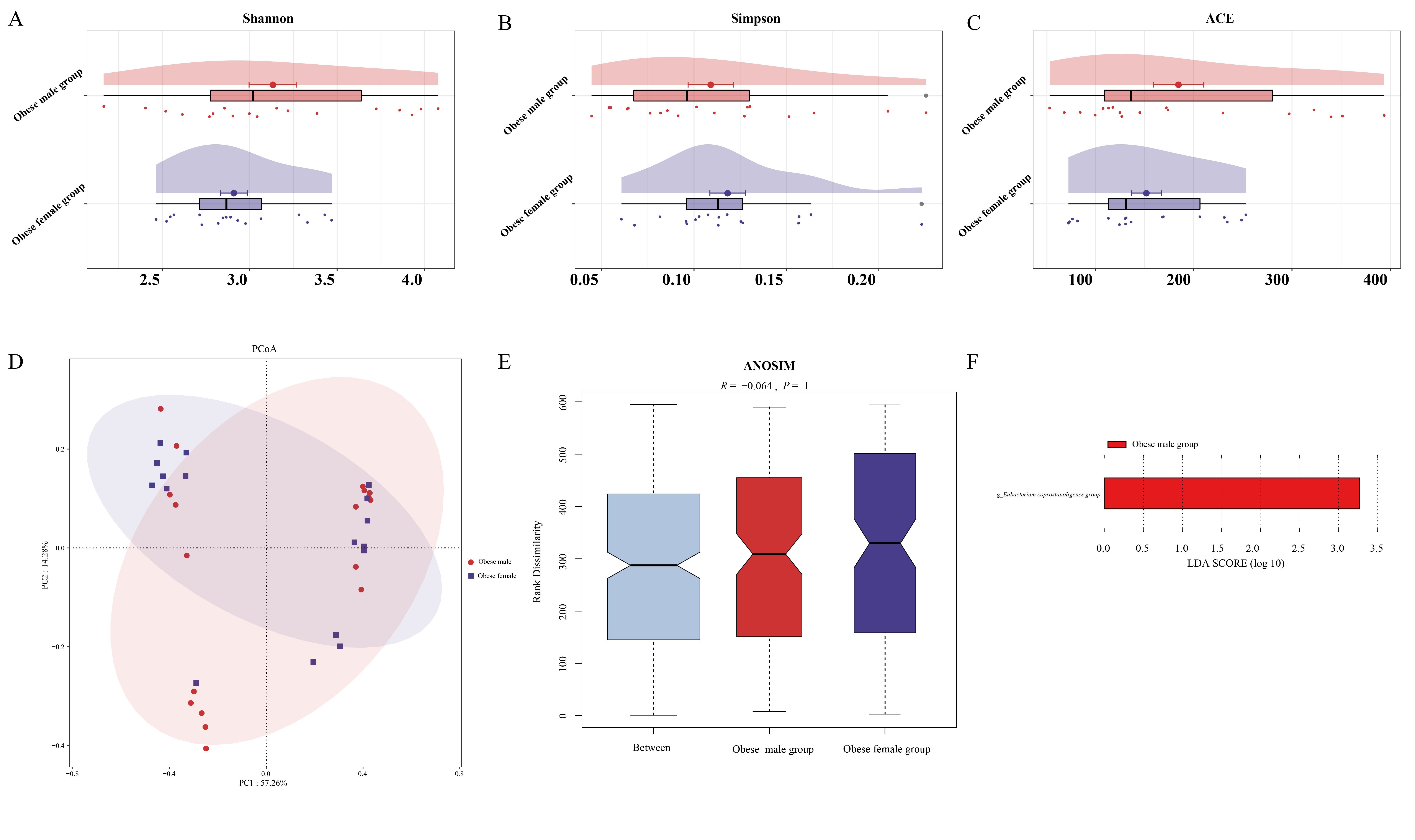

Supplement: Supplementary file 1 [file Image_1.tif]

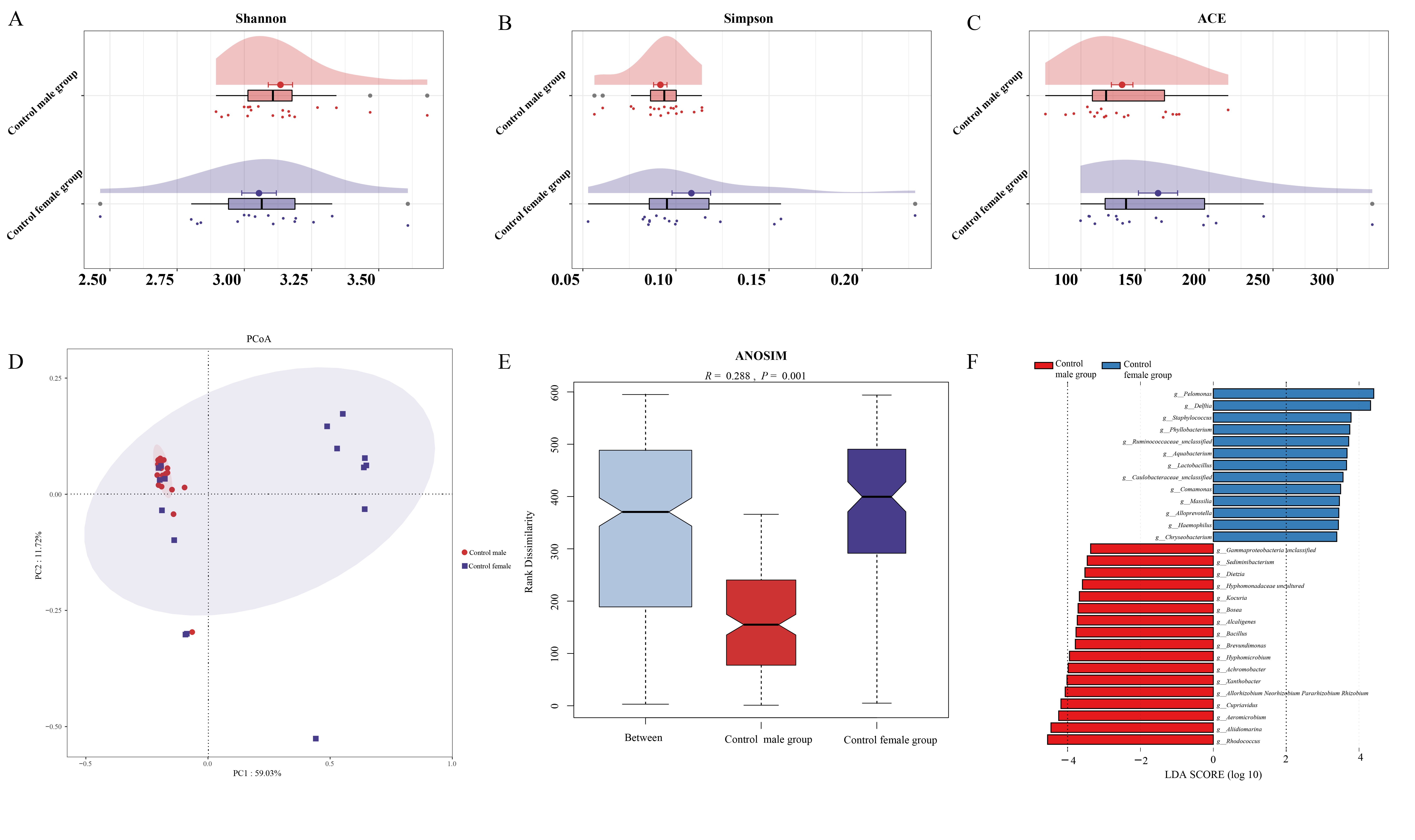

Supplement: Supplementary file 2 [file Image_2.tif]
