# Supplementary material for: Altered ocular surface microbiota in obesity: a case-control study
Source: Front Cell Infect Microbiol. 2024 Mar 12;14:1356197. doi: 10.3389/fcimb.2024.1356197 (PMC10963539; doi:10.3389/fcimb.2024.1356197)
Supplement: Supplementary file 3 [file Table_1.docx]

**SUPPLEMENTARY TABLE 1 Statistics of alpha diversity.**

| Term | Obese group | Healthy group | *P* value |
| --- | --- | --- | --- |
| Shannon index | 2.906 (2.719, 3.305) | 3.089 (2.999, 3.187) | 0.100 |
| Simpson index | 0.108 (0.082, 0.130) | 0.094 (0.086, 0.102) | 0.143 |
| ACE index | 131.218 (110.482, 230.517) | 128.3488(110.6796,169.0159) | 0.506 |
